# Knowledge and awareness of ocular allergy among undergraduate students of public universities in Ghana

**DOI:** 10.1186/s12886-016-0366-2

**Published:** 2016-10-28

**Authors:** Samuel Kyei, Bernard Tettey, Kofi Asiedu, Agnes Awuah

**Affiliations:** 1 Department of Optometry, School of Allied Health Sciences, College of Health and Allied Sciences, University of Cape-Coast, Cape-Coast, PMB Ghana; 2 Refraction and Low Vision Clinic, Eye Center, Komfo Anokye Teaching Hospital, P. O Box 1934, Kumasi, Ghana

**Keywords:** Conjunctivitis, Knowledge, Ocular allergy, Allergen, Assessment

## Abstract

**Background:**

Ocular allergy is a growing public health problem that greatly impacts the day-to-day life of sufferers and their families. Other aspects of their activities of daily living such as schooling, professional, and social life are affected hence an increased awareness and knowledge of ocular allergies, their detection and treatment is paramount. This study was to assess the level of knowledge and awareness of ocular allergy among undergraduate students of public universities in Ghana.

**Methods:**

A descriptive cross sectional survey was conducted among 1000 students from three selected public universities in Ghana. Each respondent completed a questionnaire that had questions concerning awareness and knowledge of ocular allergy.

**Results:**

Out of the 1000 students, 347 (34.7 %) were aware of ocular allergy. Of these 347 students, the level of knowledge of ocular allergy was generally low. Majority of the students had their source of information about ocular allergy from the media and the internet. There was statistical significant association among awareness of ocular allergy, sources of information and programme of study (*p* < 0.001).

**Conclusion:**

Level of awareness among university students is generally low. Students’ programmes of study influenced their knowledge of ocular allergy. Public health measures are recommended to help educate students on the prevention and control of ocular allergy as well as the complications associated with this condition.

## Background

Ocular allergy also called allergic conjunctivitis represents a group of hypersensitivity disorders where the eyes produce an abnormal immunological response to normally harmless antigens (allergens) resulting in symptoms such as itching, tearing, burning, foreign-body sensation and ocular dryness. These symptoms of ocular allergy can begin at any age but children and young adults are the most susceptible. Allergies in general constitute the third most chronic disorder among children and ocular manifestation (allergic conjunctivitis) is the primary reason for pediatric eye consult [1–3]. The last four decades have actually seen an exponential increase in allergic diseases with about 15–20 % of the world’s population suffering ocular allergies alone [4–6]. It is reported that up to 40 % of people of all age groups across the globe are affected by allergic diseases in its various forms [7]. A single reason for this upsurge in allergic diseases cannot be pinpointed. Experts are therefore contemplating the contribution of numerous factors, including genetics, air pollution, pets, and early childhood exposure [8]. Most of these factors are environmental resulting mainly from increasing industrialization [9].

In Ghana, the prevalence of allergic conjunctivitis from a hospital based study was found to be 9.1 % (1718 out of 18,896 patients) [10]. However, other community based studies among school children indicated a higher prevalence of 17.3 and 39.9 % respectively [11, 12]. Not only is it prevalent among children but several other studies have reported relatively higher prevalence than cataract and glaucoma among adults in Ghana [13, 14] yet it has not received the necessary attention accorded these other ocular disorders [15].

Other studies elsewhere in Hong Kong and France involving pediatric patients report that up to 30 and 32 % of children with allergies respectively had ocular symptoms as the sole manifestation of their allergic disorder [12, 16, 17]. This makes ocular allergy one of the most common ocular diseases encountered in clinical practice, especially among children. Allergic disease involving the eye in the form of allergic rhinoconjunctivitis is not only common but a disease of public health concern due to its impact on quality of life and economic cost [18]. It is clearly evident that ocular allergy interferes with activities of daily living, particularly, academic, sleep, driving, extracurricular and other social activities of sufferers [18, 19].

The chronic forms of allergic diseases involving the eye (the lids, conjunctivae, and corneas) such as vernal keratoconjunctivitis (VKC), atopic keratoconjunctivitis (AKC), and giant papillary conjunctivitis (GPC) [12, 16] may necessitate co-management with allergists, dermatologists, and pediatricians. This poses a cost burden of specialists’ consultation to sufferers [20]. For instance, the estimated cost burden in treating ocular allergy in the United States alone is approximately 5.9 billion dollars [21]. These chronic forms may also induce ocular surface tissue remodelling as there are no safe, long-term treatment regimens for severe forms of ocular allergic diseases.

However, pharmacological management is beset with challenges such as varying efficacy from patient to patient, drug cost, and potential complications such as dry eye, cataract and glaucoma [10]. This highlights the need for in-depth public health measures after careful assessment of the awareness and knowledge level of various populations. There is an urgent need to prioritize and concert research efforts in the field of ocular allergy. This will help in achieving sustainable results in prevention and control of this prevalent and chronic disease of the 21^st^century [22]. This is even moreso, as the long term pharmacological treatment with steroids and antihistamines; the mainstay drugs used in Ghana have been linked with complications such as glaucoma, cataract and dry eye disease [10, 20, 23–25].

Various population-based studies have shown that awareness and knowledge of other eye diseases such as glaucoma and cataract among both rural and urban populations is low in developed countries and worse in developing countries [26–36]. Nevertheless, it is acknowledged that greater awareness of the allergic conjunctivitis and knowledge of treatment options will contribute immensely to the management of ocular allergy as a synergy to the effort of eye care practitioners [37]. Students in universities are better placed as agents of change in their communities and their knowledge of ocular allergy will not only impact their quality of life but their communities’ at large and informed public health policies. This study therefore, sought to assess the knowledge and awareness of ocular allergy among university students in selected public universities in Ghana.

## Methods

### Study design

A descriptive cross-sectional study using a pre-tested semi-structured questionnaire was conducted among university students in three major public universities in Ghana. The questionnaire was designed based on a review of related studies [36, 38]. The questionnaire covered three main areas; demographic details, awareness and knowledge of ocular allergy. Awareness as used in this study was defined as ‘having heard of ocular allergy’ and consisted of several questions on basic national and international epidemiological facts aimed at establishing their awareness of ocular allergy. The questions about knowledge of ocular allergy centered on the definition of ocular allergy, signs and symptoms, common triggers of the disorder, as well as general treatment and management protocols.

### Data collection procedure and sampling technique

The three universities out of the 8 public universities were conveniently selected in Ghana as these selected universities run the widest range of programmes and adequately represent students from all 10 political regions of the Country. These 3 institutions together had registered regular students (apart from those on distance learning) population of 87,224 at the time of the study. All these universities were organized into collegiate system. University of Ghana, (UG) structured into four colleges (Basic and Applied Sciences, Education, Health Sciences and Humanities) had a population of 29,754. Kwame Nkrumah University of Science and Technology, (KNUST) with six colleges (Agricultural and Natural Resources, Health Sciences, Art and Social Sciences, Architecture and Planning, Engineering, and Sciences) had 38,439 students. University of Cape Coast, (UCC) with five colleges (Agricultural and Natural Sciences, Distance Education, Education Studies, Humanity and Legal Studies, Health and Allied Sciences) had 19,031 students. A total of 1300 questionnaires were proportionately allotted to these 3 universities according to their population sizes. UG was allotted 443, KNUST; 573 and UCC; 284 of the questionnaires. Based on the assumptions that students were evenly distributed across the various colleges, the allotted questionnaires to each university were equally assigned to the colleges of the universities. A list of students in each college was then obtained from the colleges’ registry and every 50^th^ student was selected. The selected students were approached with addressed questionnaires by a team of trained research assistants. The teams of assistants were recruited from each university campus as they were familiar with the terrain on their respective campuses. The data collection lasted for a period of 3 months. The retrieved completed questionnaires were then sorted for each university and the following groupings were derived: Sciences (biological, physical, mathematical, agricultural, engineering sciences, etc.); Arts, Social Sciences and Law; Education; Business (Accounting, management, commerce, business administration, etc); and Health Sciences (medicine, optometry, dentistry nursing, pharmacy, physiotherapy, medical laboratory technology, herbal medicine, veterinary medicine, etc.).

### Data analysis

The data obtained were entered into the Statistical Product and Service Solutions (SPSS version 21). The same tool was used for data analysis. Descriptive statistics, frequency tables and percentages were used in the data analysis and interpretation. The relationship between awareness of ocular allergy and demographic factors such as age, gender, level, and programme of study was assessed using the chi-square test. A *p*-value of less than 0.05 was considered statistically significant.

## Results

### Demographic characteristics

Twenty questionnaires were pretested among 20 non-optometry students for the corrections of ambiguities and minimization of medical jargons. The questionnaire was then modified and pretested again among 50 non health science students for further revision and adjustment before final field administration. Of the 1300 questionnaires that were distributed, 1000 completed questionnaires were retrieved, giving an overall response rate of 76.9 %. The details with respect to the 3 universities are as follows: UG (341 out of 443, 77.0 %), KNUST (441 out of 573, 71.7 %) and UCC (218 out of 284, 76.8 %). The respondents included 605 males (60.5%). Their ages ranged from 17 to 40 years with a mean age of 22 ± 2.32 years (Table 1).

**Table 1:** Demographic characteristics

Gender	Male	Female	Total	
	605 (60.5 %)	395 (39.5 %)	1000	
Age	17–25 years	Above 25 years	Total	
	949 (94.9 %)	51 (5.1 %)	1000	
Programmes	UG	KNUST	UCC	Total
Sciences	65	146	27	238
Arts	134	124	32	290
Education	_	_	100	100
Business	60	55	34	149
Health Sciences	82	116	25	223

### Awareness of ocular allergy

Out of the 1000 participants, only 347 were aware of ocular allergy as per the operational definition, citing varying sources as “where they heard about ocular allergy”. These sources were: medical training, media, internet, a relative or friend and from an eye care practitioner. The media was the source of awareness and knowledge among 82 (23.6 %) of the participants. The next major source of information about ocular allergy was the internet which was the source of 81 (23.3 %) of the respondents. Fifty eight (16.7 %) participants acquired their awareness and knowledge from their medical training. This was same (*n* = 58, 16.7 %) for respondents whose source of awareness and knowledge was from Eye care practitioners. Two hundred and eight (59.9 %) respondents (out of the 347) knew that millions of people worldwide are affected by ocular allergies each year while 61.4 % were aware that ocular allergy could lead to visual loss. Only 32.0 % were aware of the fact that about forty percent of Ghanaians suffer from ocular allergy. Out of the 347 respondents, 152 (43.8 %) were not aware that Ocular allergy is one of the most common eye conditions encountered by eye care professionals as well as General practitioners. However, only 85.9 % of the respondents were aware of itching, as a primary source of discomfort among those who suffer ocular allergies. An association was found between the institution and awareness of ocular allergy (*χ* ^2^ = 16.130, df =2, *p* < 0.001). Again, an association was found between the programme of study and the awareness of ocular allergy (*χ* ^2^ = 112.372, df =4, *p* < 0.001). This is shown in Table 2.

**Table 2:** Awareness of Ocular Allergy

	Programme of study						
Parameter		Science	Humanities	Education	Business	Health sciences	Total
What is your source of information	Media	25 (26.6)	28 (27.5)	9 (47.4)	5 (29.4)	15 (13.0)	82 (23.6)
	Internet	22 (23.4)	26 (25.3)	3 (15.8)	5 (29.4)	25 (21.7)	81 (23.3)
	Friends/relatives	17 (18.1)	17 (16.7)	2 (10.5)	4 (23.5)	14 (12.2)	54 (15.6)
	Eye specialist	13 (13.8)	14 (13.7)	3 (15.8)	3 (17.6)	25 (13.0)	58 (16.7)
	Program training	13 (13.8)	11 (10.8)	2 (10.5)	0	32 (27.8)	58 (16.7)
	Others	4 (4.3)	6 (5.9)	0	0	4 (3.5)	14 (4.0)
In your opinion does ocular allergy affect millions of people worldwide each year?	No	8 (8.5)	9 (8.8)	1 (5.3)	1 (5.9)	11 (9.6)	30 (8.6)
	Yes	64 (68.1)	61 (59.8)	11 (57.9)	10 (58.8)	62 (53.9)	208 (59.9)
	Don’t know	22 (24.4)	32 (31.4)	7 (36.8)	6 (35.3)	40 (34.8)	107 (30.8)
About forty percent of the Ghanaian population suffers from ocular allergy	True	34 (36.2)	35 (34.3)	5 (26.3)	7 (41.2)	30 (26.1)	111 (32.0)
	False	7 (7.4)	6 (5.9)	3 (15.8)	1 (5.9)	8 (7.0)	25 (7.2)
	Don’t know	53 (56.4)	51 (50.0)	11 (57.9)	9 (52.9)	77 (67.0)	211 (60.8)
Could ocular allergy lead to visual loss?	No	19 (20.2)	13 (12.7)	3 (15.8)	3 (17.6)	23 (20.0)	61 (17.6)
	Yes	62 (66.0)	69 (67.6)	14 (73.7)	13 (76.5)	55 (47.8)	213 (61.4)
	Don’t know	13 (13.8)	20 (19.6)	2 (10.5)	1 9 (5.9)	37 (32.2)	73 (21.0)
Ocular allergy is one of the most common eye conditions encountered by General Practitioners as well as eye care practitioners	True	55 (58.5)	51 (50.0)	7 (36.8)	8 (47.1)	74 (64.3)	195 (56.2)
	False	6 (6.4)	6 (5.9)	0	1 9 (5.9)	14 (12.2)	27 (7.8)
	Don’t know	33 (35.1)	45 (44.1)	12 (63.2)	8 (47.1)	27 (23.5)	125 (36.0)
Majority of people who have ocular allergy experience itching as the primary source of discomfort	True	84 (89.4)	80 (78.4)	13 (68.4)	13 (76.5)	108 (93.9)	298 (85.9)
	False	3 (3.2)	6 (5.9)	1 (5.3)	2 (11.8)	1 (0.1)	13 (3.7)
	Don’t know	7 (7.4)	16 (15.7)	5 (26.3)	2 (11.8)	6 (5.2)	36 (10.4)

### Knowledge of ocular allergy

Out of the 347 respondents who were aware, 194 representing 55.9 % defined ocular allergy as inflammation of the eye in response to a non-infectious or harmless foreign agent entering the eye. Sixty-six respondents, representing 19.0 % defined ocular allergy as discomfort and pains within the eye. Fifty-five respondents, representing 15.9 % defined ocular allergy as an eye condition caused by frequent rubbing of the eye. Thirty-two (9.2 %) did not know what ocular allergy was. Out of the 347 respondents, 39 (11.2 %) participants believed that ocular allergy affects the conjunctiva only. Twenty -two (6.3 %) participants believed that ocular allergy affects the cornea only. One hundred (22.8 %) respondents knew that ocular allergy affects the conjunctiva, cornea and eyelids, while 64 (18.4 %) respondents knew that ocular allergy affects the conjunctiva, cornea, limbus, and eyelids. However, 122 (35.2 %) did not know the eye structures involved in ocular allergy (Table 3).

**Table 3:** Knowledge of ocular allergy

	Programme of study						
Parameter		Science	Humanities	Education	Business	Health sciences	Total
What is your understanding of ocular allergy?	Caused by frequent rubbing of the eye	19 (20.2)	17 (16.7)	6 (31.6)	5 (29.4)	8 (7.0)	55 (15.8)
	Inflammation of the eye in response to harmless foreign substance	46 (48.9)	48 (47.1)	7 (36.8)	4 (23.5)	89 (77.4)	194 (55.9)
	Discomfort and pains within the eye	23 (24.5)	24 (23.5)	2 (10.5)	4 (23.5)	13 (11.3)	66 (19.0)
	Don’t know	6 (6.4)	13 (12.7)	4 (21.1)	4 (23.5)	5 (4.3)	32 (9.2)
Eye structures involved in ocular allergy include	Conjunctiva	11 (11.7)	12 (11.7)	3 (15.80	3 (17.6)	10 (8.7)	39 (11.2)
	Cornea	10 (10.6)	3 (2.9)	4 (21.1)	2 (11.8)	3 (2.6)	22 (6.3)
	Conjunctiva, cornea, eyelid	25 (26.6)	21 (20.6)	0	6 (35.3)	48 (41.7)	100 (28.8)
	Conjunctiva, cornea, limbus,eyelid	13 (13.8)	15 (14.7)	1 (5.3)	2 (11.8)	33 (28.7)	64 (18.4)
	Don’t know	35 (37.2)	51 (50.0)	11 (57.9)	4 (23.5)	21 (18.30	122 (35.2)
Are ocular allergies transmitted from person to person?	Yes	64 (68.1)	55 (53.9)	8 (42.1)	11 (64.7)	77 (67.0)	215 (62.0)
	No	8 (8.5)	10 (9.8)	1 (5.3)	1 9 (5.9)	11 (9.6)	31 (8.9)
	Don’t know	21 (22.3)	37 (36.3)	10 (52.6)	5 (29.4)	27 (23.5)	100 (28.8)
What is the best way to prevent ocular allergy?	Apply anti- allergic drugs	6 (6.4)	9 (8.8)	2 (10.5)	5 (29.4)	13 (11.3)	35 (10.1)
	Stay away from those with the disease	1 (1.1)	4 (3.9)	1 (5.3)	0	5 (4.3)	11 (3.2)
	Avoid the source of trigerrs	52 (55.3)	47 (46.1)	6 (31.6)	6 (35.3)	83 (72.2)	194 (55.9)
	Visit eye clinics	24 (25.5)	30 (29.4)	7 (36.8)	4 (23.5)	8 (7.0)	73 (21.0)
	Don’t know	11 (11.7)	12 (11.7)	3 (15.8)	2 (11.8)	5 (4.3)	33 (9.5)
Do you know substances that trigger allergies in the eye?	No	52 (55.3)	69 (67.6)	15 (78.9)	11 (64.7)	54 (47.0)	201 (57.9)
	yes	42 (44.7)	33 (32.4)	4 (21.1)	6 (35.3)	61 (53.0)	146 (42.1)
Do you know of medications used for treating ocular allergy?	No	77 (81.9)	86 (84.3)	17 (89.5)	14 (82.4)	79 (68.7)	273 (78.7)
	yes	17 (18.1)	16 (15.7)	2 (10.5)	3 (17.6)	36 (31.3)	74 (21.3)
Some eye allergies are inherited	True	44 (46.8)	57 (55.9)	11 (57.9)	10 (58.8)	46 (40.0)	168 (48.4)
	False	13 (13.8)	12 (11.7)	3 (15.8)	1 (5.9)	19 (16.5)	48 (13.8)
	Don’t know	37 (39.4)	33 (32.3)	5 (26.3)	6 (35.3)	50 (43.5)	131 (37.8)
Smoking is a risk factor for ocular allergy	True	49 (52.1)	48 (47.1)	10 (52.6)	11 (64.7)	54 (47.0)	172 (49.6)
	False	8 (8.5)	7 (6.9)	0	1 (5.9)	11 (9.6)	27 (7.8)
	Don’t know	37 (39.4)	47 (48.0)	9 (47.40	5 (29.4)	50 (43.5)	148 (42.7)

Out of the 347 respondents, 300 (86.5 %) respondents recognized ‘itching’ as a symptom, 277 (79.8 %) respondents identified ‘redness’ as a sign, while 153 (44.1 %) respondents recognized ‘discharge’ as a sign. One hundred and eighty-one (52.2 %) respondents ticked ‘tearing’ as a symptom, 91 (26.2 %) recognized ‘photophobia’ as a symptom while 147 (42.4 %) respondents identified ‘blurred vision’ as a symptom of ocular allergy. Two hundred and forty-one (69.5 %) respondents knew that ‘headache’ is not a symptom of ocular allergy while 294 (84.7 %) respondents were able to recognize that ‘nausea’ is not a symptom (Table 4). Out of the 347 respondents, only 97 (28.0 %) respondents recognized ‘seasonal allergic conjunctivitis’ as a form of ocular allergy, 43 (12.4.8 %) respondents identified ‘perennial allergic conjunctivitis’ as a form, while 50 (14.4 %) respondents recognized ‘vernal conjunctivitis’ as a form of ocular allergy. Twenty-eight (8.1 %) respondents ticked ‘giant papillary conjunctivitis’ as a form, and 38 (11.0 %) respondents recognized ‘atopic keratoconjunctivitis’ as a form of ocular allergy. Seventy-eight (22.5 %) respondents knew that ‘bacterial conjunctivitis’ is not a form of ocular allergy while 117 (33.7 %) respondents were able to recognize that ‘viral conjunctivitis’ is not a form of ocular allergy (Table 5).

**Table 4:** Knowledge of signs and symptoms of ocular allergy

	Frequency (Percentages)		
Signs and symptoms	Yes	No	Don’t know
Redness	277 (79.8)	43 (12.4)	27 (7.8)
Headache	5 (21.6)	241 (69.5)	31 (8.9)
Itching	300 (86.5)	21 (6.1)	26 (7.5)
Nausea	18 (5.2)	294 (84.7)	35 (10.1)
Discharge	153 (44.1)	163 (46.9)	31 (9.0)
Blurred vision	147 (42.4)	167 (48.1)	33 (9.5)
Photophobia	91 (26.2)	224 (64.6)	32 (9.2)
Tearing	181 (52.2)	136 (39.2)	30 (8.6)

**Table 5:** Knowledge of the forms of ocular allergy

Forms of ocular allergy	Frequency (percentages)		
	Yes	No	Don’t know
Bacterial conjunctivitis	102 (29.4)	78 (22.5)	167 (48.1)
Seasonal conjunctivitis	97 (28.0)	83 (23.9)	167 (48.1)
Viral conjunctivitis	61 (17.6)	117 (33.7)	169 (48.7)
Giant papillary	28 (8.1)	150 (43.2)	169 (48.7)
Vernal conjunctivitis	50 (14.4)	130 (37.5)	167 (48.1)
Perennial conjunctivitis	43 (12.4)	136 (39.2)	168 (48.4)
Atopic keratoconjunctivitis	38 (11.0)	140 (40.3)	169 (48.7)

Thirty-five respondents, representing 10.1 % indicated that best way of preventing ocular allergy is to apply anti-allergic medication. Eleven respondents, representing 3.2 % said that staying away from those having ocular allergy was the best way to prevent ocular allergy. One hundred and ninety-four respondents, representing 55.9 % knew that avoiding the source of allergy triggers was the best way to prevent ocular allergy. Seventy-three (21.0 %) responded that the best way of preventing ocular allergy is to visit eye clinics. Thirty-three (9.5 %) did not know how ocular allergies could be prevented. Out of the 347 participants, 146 (42.1 %) knew of substances which trigger allergic response in the eye while 201 (57.9 %) did not know of substances which trigger allergic response in the eye. Respondents who knew of ocular allergy triggers were made to specify them. Multiple responses were allowed since the question was an open-ended one and 74 out 347 respondents representing 21.3 % could mention at least one drug used in management of ocular allergy. The remaining 273 participants representing 78.7 % had no knowledge of drugs used in managing ocular allergy.

Out of the 347 participants, 31 (8.9) respondents indicated that ocular allergy is transmitted from one person to another; 215 respondents knew that ocular allergy is not transmitted from person to person and 101 (29.1) respondents had no knowledge about it. Also 168 (48.4 %) said that ocular allergies are inherited, while 48 (13.8 %) said this assertion was false. One hundred and thirty-one respondents representing 37.8 % did not know whether this assertion was true or false. Again, 172 (49.6 %) said that smoking is a risk factor for ocular allergy while 27 (7.8 %) said the assertion was false. One hundred and forty-eight respondents representing 42.7 % did not know whether this assertion was true or false (Table 3).

## Discussion

### Awareness of ocular allergy

The study was based on the assumptions that students were evenly distributed across the colleges which could affect the representativeness of the various constituents of the universities. The questionnaire used has the limitation of ascertaining the truthfulness of responses provided. Nevertheless, the large size of the study sample has the benefit of mitigating these limitations to some extent.

The awareness level of ocular allergy among students at the tertiary level was relatively low. Ocular allergy is not considered a top priority eye disease and has not received adequate public health attention despite its noisome symptoms and complications [39]. This could have accounted for the low awareness level. Besides this, reports regarding awareness of some of the priority eye diseases such as glaucoma and cataract have been found to be low, raising concern for intense public education [26–36]. Similarly, another study found generally low awareness level among patients with respect some emergent ophthalmic diseases such as retinal detachment, acute angle-closure glaucoma, giant cell arteritis, and central retinal artery occlusion [40]. This obviously has implication for early medical presentation and maximization of visual prognosis. Awareness level of eye diseases is generally low compared to sexually transmitted infections, STIs and coronary heart diseases [41, 42].

It is worthy to note that half of students (51.6 %) who were aware of ocular allergy were from the health sciences indicating the extent of ignorance among the educated non health science students. The health science students heard of ocular allergy mainly through their medical training as well as contact with eye care providers most of whom are involved with their training. This presupposes that public education on ocular allergy is either non-existing or rudimentary among university students in Ghana. Programme of study, and for that matter area of specialty, was therefore a useful predictor of awareness of ocular allergy.

### Knowledge of ocular allergy

Out of the 347 participants who were aware of ocular allergy, more than half (55.9 %) could correctly define ocular allergy as inflammation of the eye in response to a non-infectious or when a harmless foreign agent make contact with the eye. Such students were mainly from the health sciences (45.9 %) and the sciences (23.7 %). Some 44.1 % from education, business and arts did not know the definition for ocular allergy. Thus health science students had the advantage of having good knowledge about ocular allergy over the non-health science students.

The findings of this study indicate that itching (86.5 %) was the most recognized symptom of ocular allergy among the respondents who were aware of the condition unlike the reported poor knowledge of symptoms of giant cell arteritis, and central retinal artery occlusion [40]. Itching is typical of ocular allergy and diagnosis without the symptom of ocular itch is always problematic [10, 15, 21]. Studies have shown that bothersome nature of ocular itch is a major drive for the practice of ocular self medication not only among university students [43] but the general population [44]. Photophobia (26.2 %) was the least recognized symptom of ocular allergy as it associated with several other ocular diseases other than allergy [45]. In the study, more than half (57.9 %) did not know of agents which trigger allergic response in the eye. This underscore the essence of public health education since knowledge of allergy triggers is key to the prevention and control of ocular allergy [38, 46]. However, more than half (55.9 %) knew that avoiding the source of allergy triggers was the best way to prevent ocular allergy. Avoidance of allergens is the ideal non-pharmacological approach to the control and prevention of allergic eye disease despite the challenges it poses due to the lack of awareness on the distributions and density of these widespread triggers among the populace [20]. Most of the students who knew this acquired their source from medical training, eye care professionals and the internet. Dust was the most recognized trigger of ocular allergy followed by pollen, smoke and pet dander respectively. These allergens identified by the respondents have been found to be common in the tropics and have particularities of interest as they provide clues to improved understanding of the pathogenesis, diagnosis and management of allergic diseases in the Tropics [47].

Out of the 347 respondents who were aware of the disorder, less than one-quarter (21.3 %) could mention at least one drug used in management of ocular allergy, the remaining 273 three-quarters (78.7 %) had no knowledge of drugs used in managing ocular allergy given the fact that most of these ophthalmic preparations for allergy are sold over the counter. This presupposes that they are unlikely not to risk self medication or advice pharmacological intervention in case ocular allergy since they have little knowledge. However, should they risk the use of this option it could lead to all sort of adverse reactions as a result of their unformed actions or inactions [48].

### Source of awareness and knowledge about ocular allergy

In this study, majority of the respondents acquired their knowledge of ocular allergy through the media and the internet. Medical training and information from eye care providers played the second major role in creating awareness of among 33.4 % of the 347 respondents. Information from relatives or friends on ocular allergy contributed similarly to awareness creation. This highlights the need for an informed community capable of the provision of timely basic education at the primary care level. There was a statistically significant association between the programme of study and the source of information, which is consistent with other studies among hospital workers where different workers with different area specialty had had different sources of information [35]. It can be deduced from the study that the media was the major source of information among the arts, education and business and science students. Respondents who had their knowledge of ocular allergy through medical training as well as eye care providers tended to have more knowledge than those who chose media and internet as their main source of information. There was a lag in knowledge of ocular allergy acquired from the media compared to that acquired during the course of medical training. Thus though the media was the leading source in terms of ocular allergy awareness creation, people who chose the media demonstrated the weakest level of knowledge about ocular allergy. It is not clear exactly what the media conveys to the general public about ocular allergy. This gives a clue that perhaps there is a lot of public misinformation about diseases from the media. It is questionable which resource persons’ champion the course of public education through the media as this seems to the trend in other awareness and knowledge studies [41, 49].

## Conclusions

From the study, it can be concluded that the level of awareness of ocular allergy was relatively low among university students and far lower when students from the health sciences were excluded. Though the media was the leading source in terms of awareness creation, students who had their source from the media had the weakest level of knowledge of ocular allergy. Students who had their knowledge through medical training and eye care providers demonstrated the highest level of knowledge of ocular allergy.

Therefore extra effort should be made by relevant stakeholders in creating awareness in order to reduce the impact of ocular allergy on the quality of life of students and their potential complications.

## Abbreviations

AKC: Atopic keratoconjunctivitis
GPC: Giant papillary conjunctivitis
KNUST: Kwame Nkrumah University of Science and Technology
SPSS: Statistical product and service solutions
UCC: University of Cape Coast
UG: University of Ghana
VKC: Vernal keratoconjunctivitis
